# Characterization of a *Fusarium graminearum* Salicylate Hydroxylase

**DOI:** 10.3389/fmicb.2018.03219

**Published:** 2019-01-08

**Authors:** Guixia Hao, Todd A. Naumann, Martha M. Vaughan, Susan McCormick, Thomas Usgaard, Amy Kelly, Todd J. Ward

**Affiliations:** Mycotoxin Prevention and Applied Microbiology Research Unit, National Center for Agricultural Utilization Research, United States Department of Agriculture – Agricultural Research Service, Peoria, IL, United States

**Keywords:** *Fusarium graminearum*, salicylate hydroxylase, Fusarium head blight, mutagenesis, heterologous expression

## Abstract

Salicylic acid (SA) plays an important role in regulating plant defense responses against pathogens. However, pathogens have evolved ways to manipulate plant SA-mediated defense signaling. *Fusarium graminearum* causes Fusarium head blight (FHB) and reduces crop yields and quality by producing various mycotoxins. In this study, we aimed to identify the salicylate hydroxylase in *F. graminearum* and determine its role in wheat head blight development. We initially identified a gene in *F. graminearum* strain NRRL 46422 that encodes a putative salicylate hydroxylase (designated *FgShyC*). However, the *FgShyC* deletion mutant showed a similar ability to degrade SA as wild-type strain 46422; nor did overexpression of FgShyC in *E. coli* convert SA to catechol. The results indicate that *FgShyC* is not involved in SA degradation. Further genome sequence analyses resulted in the identification of eight salicylate hydroxylase candidates. Upon addition of 1 mM SA, FGSG_03657 (designated *FgShy1*), was induced approximately 400-fold. Heterologous expression of FgShy1 in *E. coli* converted SA to catechol, confirming that FgShy1 is a salicylate hydroxylase. Deletion mutants of *FgShy1* were greatly impaired but not completely blocked in SA degradation. Expression analyses of infected tissue showed that *FgShy1* was induced during infection, but virulence assays revealed that deletion of FgShy1 alone was not sufficient to affect FHB severity. Although the *Fgshy1* deletion mutant did not reduce pathogenicity, we cannot rule out that additional salicylate hydroxylases are present in *F. graminearum* and characterization of these enzymes will be necessary to fully understand the role of SA-degradation in FHB pathogenesis.

## Introduction

Phytohormones including salicylic acid (SA) and jasmonic acid (JA) play key roles in activation of pathogen-associated molecular pattern (PAMP)-triggered immunity (PTI), effector-triggered immunity (ETI), and triggering systemic acquired resistance (SAR) (An and Mou, 2011). Generally, SA contributes to plant defense against biotrophic and hemibiotrophic pathogens whereas JA promotes disease resistance against necrotrophic pathogens (Glazebrook, 2005). The importance of SA-mediated defense has been demonstrated in *Arabidopsis* and *Nicotiana*. Transgenic plants expressing the bacterial gene *NahG* for salicylate hydroxylase, which degrades SA, are more susceptible to several pathogens (Gaffney et al., 1993). In *Arabidopsis*, SA induction-deficient (sid) mutants, which have an inactivated isochorismate synthase gene, fail to synthesize SA in response to pathogen infection, resulting in enhanced susceptibility to bacterial and fungal pathogens (Nawrath and Métraux, 1999; Wildermuth et al., 2001). *Nicotiana benthamiana* plants treated with SA are more resistant to *Agrobacterium* infection. Furthermore, silencing of genes involved in SA biosynthesis and signaling in *N. benthamiana* increases susceptibility to *Agrobacterium* infection (Anand et al., 2008).

Because SA-mediated signaling plays a critical role in plant defense, phytopathogens have developed various strategies to interfere with the plant SA biosynthetic pathway. *Ustilago maydis* secretes chorismate mutase (Cmu1), which depletes the SA precursor chorismate, and thereby reduces plant SA biosynthesis and accumulation (Djamei et al., 2011). *Phytophthora sojae* and *Verticillium dahliae* secrete isochorismatases that hydrolyze the SA precursor isochorismate, resulting in suppression of the SA pathway and host immunity (Liu et al., 2014). In bacteria, a salicylate hydroxylase in *Ralstonia solanacearum* degrades plant SA to protect itself from SA inhibition and to enhance its virulence on tobacco (Lowe-Power et al., 2016). The salicylate hydroxylase produced by the citrus greening pathogen ‘*Ca.* L. asiaticus’ (Las) degrades SA and suppresses plant defenses (Li et al., 2017). Although a functional salicylate hydroxylase, Shy1, was identified from the smut fungus *U. maydis*, its inactivation did not have a significant effect on smut disease (Rabe et al., 2013).

Apart from its role in plant defense responses, SA can directly affect bacterial and fungal growth. SA inhibits spore germination and colony growth of *Fusarium graminearum* and *Harpophora maydis* (Qi et al., 2012; Degani et al., 2015) and significantly reduces hyphal growth in *Aspergillus flavus* (Panahirad et al., 2014). The direct inhibition effects of SA are also found on bacteria including *Agrobacterium tumefaciens* (Anand et al., 2008) and *R. solanacearum* (Lowe-Power et al., 2016). On the other hand, exogenous SA promotes *in vitro* growth of the fungal pathogen *Moniliophthora perniciosa* (Kilaru et al., 2007). In addition, many bacteria and fungi can metabolize SA (Penn and Daniel, 2013). Salicylate hydroxylase (NahG) is well-characterized in the naphthalene degradation pathway in the bacterium *Pseudomonas putida* (You et al., 1991; Bosch et al., 1999). Salicylate hydroxylase Shy1 is required for *U. maydis* to grow on plates with SA as the sole carbon source (Rabe et al., 2013). Fungal salicylate hydroxylase enzymatic activities have been found in cell extracts of *Trichosporon cutaneum* and *Fusarium* spp. (Graminha et al., 2004; Dodge and Wackett, 2005). *Fusarium* sp. strain BI can grow with salicylate, gentisate, or catechol as the sole carbon source (Dodge and Wackett, 2005). However, salicylate hydroxylases have not been identified in *Fusarium* spp. and their potential role in Fusarium fungal–plant interactions is unclear.

*Fusarium graminearum* is the major causal agent of Fusarium head blight (FHB), which reduces wheat and barley yields and contaminates grains with the trichothecene deoxynivalenol (DON) and related mycotoxins. *F. graminearum* infection is initiated in wheat florets at anthesis, and then spreads through whole heads, facilitated by the virulence factor DON. Mutants unable to synthesize DON show FHB symptoms restricted to the initial infection sites (Proctor et al., 1995). Both SA and JA signaling are involved in FHB pathogenesis (Makandar et al., 2010; Ding et al., 2011). The role of SA in *F. graminearum* pathogenesis has been demonstrated in *Arabidopsis* using SA biosynthesis and signaling mutants (e.g., Atsid2 and Atnpr1) and transgenic plants expressing the bacterial salicylate hydroxylase NahG (Cuzick et al., 2008; Makandar et al., 2010). In addition, transgenic wheat expressing *NahG* exhibit high FHB susceptibility whereas transgenic wheat overexpressing *AtNPR1* have enhanced FHB resistance (Makandar et al., 2006, 2012). SA signaling contributes to basal defenses in wheat against *F. graminearum* (Makandar et al., 2012). However, other studies have shown that wheat heads treated with SA or a SA functional analog did not increase FHB resistance or associated PR gene expression (Yu and Muehlbauer, 2001; Li and Yen, 2008; Qi et al., 2012). Instead, SA was found to directly inhibit conidia germination and mycelial growth of *F. graminearum* (Qi et al., 2012).

Salicylic acid signaling is critical in plant defense responses. Studies suggest that coordinated and ordered expression of diverse defense signaling pathways including SA is important in FHB pathogenesis (Ding et al., 2011). Disruption of the SA defense pathway by degrading SA could play a role in disease development. Therefore, characterization of salicylate hydroxylases in *F. graminearum* will aid in elucidating the role of SA in FHB pathogenesis. In the present work, we demonstrated that *F. graminearum* can metabolize SA in culture. To determine which salicylate hydroxylase homologs of *F. graminearum* are able to degrade SA in planta and to determine the role SA degradation plays in FHB, we identified nine putative salicylate hydroxylase candidates in *F. graminearum.* We characterized FGSG_03657 (FgShy1) and demonstrate the SA-degrading ability of FgShy1 via heterologous expression and gene deletion mutagenesis. We also assessed the role of FgShy1 in *F. graminearum* pathogenesis on wheat.

## Materials and Methods

### Fungal Strains and Plants

The fungal strains used in this study include wild-type strains *F. graminearum* NRRL 46422 and PH-1. Cultures were maintained on V8 agar with a 12:12 h light/dark cycle at 28°C with ultraviolet light. Spring wheat, the susceptible Norm cultivar, was grown in controlled growth chambers under a 16:8 h light/dark cycle at 23:20°C with 50% humidity.

### Bioinformatics Analysis

The amino acid sequence of FgShyC (GenBank Accession No. BK010685), which was identified from our previous study, was used to search for SA hydroxylase homologs in the genome of *F. graminearum* PH-1 by Basic local alignment search tools (pBLAST). Eight salicylate hydroxylase candidates, FGSG_03657 (FgShy1), FGSG_09063, FGSG_00092, FGSG_10612, FGSG_05063, FGSG_04776, FGSG_10643 and FGSG_08116, were identified. Shy1 from *U. maydis* (Accession No. XM_756284) and NahG from *P. putida* (Accession No. J05317) were also included in the analysis. Sequence alignment was performed and a phylogenic tree was constructed using the MEGA7 program (Kumar et al., 2016) with the Maximum likelihood method (Whelan and Goldman, 2001) using a bootstrap value of 1000.

### SA Degradation by *F. graminearum* and Mutants

*Fusarium graminearum* NA1 strain PH-1, NA2 strain 46422, FgshyC and Fgshy1 mutants were tested for the ability to degrade SA in mung bean liquid medium. Approximately 2 × 10^5^ conidia were inoculated into 4 mL mung bean liquid medium in a 50 ml tube (Cell Treat Scientific Products, Pepperell, MA, United States). Cultures were grown in an unlit shaker (200 rpm, 28°C) for 24 h. SA was dissolved in 2% methanol with the final concentration at 100 mM and was adjusted to pH 8.0. Then 1 mM SA was added into the culture, which was grown for another 24 h in the same condition. Sterile mung bean liquid medium containing 1 mM SA in mung bean liquid medium was included for relative quantification. Since it was determined that fungal growth in mung bean medium for 24 h significantly increased the pH from 6.5 to approximately 8.3, all samples were adjusted to pH around 3.0 before SA extraction.

### SA Extraction and Analysis by GC/MS

Salicylic acid was extracted from the 4 mL cultures by adding an equal volume of HPLC grade methylene chloride (Sigma-Aldrich, St. Louis, MO, United States). As an internal standard to normalize sample extraction efficiencies, 0.1 mM of deuterated SA (3, 4, 5, 6-D4, 97%) (Cambridge Isotope Laboratories, Inc., Andover, MA, United States) was added to each sample and immediately mixed on a vortex fitted with a horizontal 15 mL tube holder for 2 min at max speed. The two phases were then separated by centrifugation at 4500 rpm for 3 min. One mL of the organic phase was transferred to a 4 mL glass vial and derivatized using 5 μL trimethylsilyldiazomethane (Sigma-Aldrich, St. Louis, MO, United States) for 30 min. Derivatization converted SA into the more volatile compounds, methyl salicylate (SA-ME) and trimethylsilyl methyl salicylate (SA-TMS), which were then collected by vapor-phase extraction and analyzed by gas chromatography/chemical ionization-mass spectrometry (GC/CI-MS) using a previously reported method (Schmelz et al., 2004; Vaughan et al., 2014). The peak areas of SA-ME and SA-TMS from each sample were summed, divided by the peak area of the deuterated SA, and then divided by area estimated from extractions of the average peak area of samples from sterile media containing 1 mM SA. Three biological replicates were analyzed for each treatment. Differences between means were determined using the Dunnett’s test which compared all other means to the sterile media containing 1 mM SA. All statistical analyses were performed with JMP statistical software (V13.1.0).

### *FgShy1* and Its Homologs Expression With SA Induction

RNA was extracted from wild-type 46422 supplemented with 1 mM SA or methanol only as control. First-strand cDNA was synthesized and reverse transcription quantitative polymerase chain reaction (RT-qPCR) was run with Bio-Rad real time polymerase chain reaction (PCR) machine. Gene expression levels were compared with the 2^-ΔΔct^ values using Bio-Rad CFX manager. Gene specific primers from *FgShyC*, *FgShy1* and salicylate hydroxylase candidates (Supplementary Table S1) were used for transcripts quantification and β-tubulin was amplified and used as internal control for gene expression normalization. For each gene, the transcripts from the methanol were set as one for calculation.

### Heterologous Expression of FgShy1

Heterologous *E. coli* strains were constructed to express FgShyC and FgShy1. The latter is identical to the theoretical protein described by NCBI reference sequence XP_011322042.1. *P. putida* NahG (You et al., 1991; UniProtKB P23262.4) was used as a positive control, as described previously (Rabe et al., 2013). Commercial expression plasmid pRSET A (Thermo Fisher) was cleaved by *NdeI* and *HindIII* restriction endonucleases. The two resulting DNAs were separated by agarose gel electrophoresis and the larger was purified. Two genes were synthesized, each encoding predicted SA hydroxylase proteins with amino-terminal GST fusions (Integrated DNA Technologies, Coralville, IA, United States). Each synthetic gene was cloned into the purified plasmid DNA by the method of Gibson (Gibson et al., 2009), using a commercial kit (New England Biolabs, Ipswich, MA, United States). Plasmid clones were verified by agarose gel analysis and DNA sequencing. Each plasmid was transformed into *E. coli* BL21 (DE3) pLYSs for expression studies and agar plate assays.

Expression studies were initiated by inoculating Luria-Bertani liquid medium (100 mL) from stationary phase cultures (1 mL inoculum). Cultures were grown in a shake incubator at 37°C for approximately 2 h (OD_600_ = 0.35). Protein expression was induced by addition of IPTG (1 mM final) and continued for 2 h. After expression, cells were precipitated from cultures by centrifugation and suspended in 0.6 mL lysis buffer (20 mM Tris-HCl, pH 8.0, 150 mM NaCl). Suspended cells were placed in 1.5 mL tubes containing 300 mg of beads (0.5 mm diameter zirconium oxide beads) and lysed in a bullet blender (Next Advance, Troy, NY, United States). Samples were analyzed by SDS-PAGE followed by whole protein staining with Coomassie stain.

### Salicylate Hydroxylase Activity Assay

Agar plate assays were performed to determine if the expressed proteins were SA hydroxylases. Expression strains were streaked from mature liquid cultures onto plates containing Luria-Bertani medium with IPTG and SA (10 g/L tryptone, 5 g/L yeast extract, 5 g/L NaCl, 15 g/L agar, 0.01 mM IPTG, 1 mM SA). Plates were incubated at 25°C and monitored for development of brown color, indicating conversion of SA to catechol, followed by its oxidation. The strain expressing *F. graminearum* FgShy1 was compared to both a positive control expressing NahG and a negative control expressing bacterial beta-galactosidase.

### Mutant Generation and Fungal Transformation

OSCAR and protoplast mediated transformation was used to create *FgshyC* and *Fgshy1* mutants following published protocols (Paz et al., 2011). A DNA fragment upstream of the start codon and a fragment downstream of the stop codon of *FgShyC* or *FgShy1* were PCR amplified from 46422 and PH-1 genomic DNA respectively with primer pairs including the BP cloning site (Supplementary Table S1). BP reactions were performed with OSCAR plasmids and products were transformed into *E. coli* competent cells. Correct constructs were confirmed by PCR analysis using two primers pairs, OSC-F and Hyg-R210, and Hyg-F850 and OSC-R (Supplementary Table S1). Then these constructs were used to obtain the fragment containing hygromycin gene (Hyg) and upstream and downstream of target gene for protoplast transformation. Hygromycin resistant isolates resulting from transformation were transferred to new V8 juice plates containing hygromycin for DNA isolation. Replacement of *FgShyC* or *FgShy1* with the hyg fragment was confirmed by PCR. Whole genome sequencing was performed to confirm the replacement of target gene with Hyg and the absence of ectopic Hyg insertion (Kelly and Ward, 2018).

### Growth Assay of *Fgshy1* Mutant

The effect of SA on the growth of the *Fgshy1* mutant was tested on 1% agar plates containing SA. Wild-type strain PH-1 served as control. Agar plates were supplemented with 0.1, 0.5, 1, or 2 mM SA from a 1 M SA stock in methanol. Plates with added methanol were used as controls. Each plate was inoculated with a plug from V8 plate. The plates were incubated at room temperature in the dark. The radial growth of mycelia was measured on day 5. Four biological replicates were performed.

To test the growth of PH-1 and *Fgshy1* mutants, 10 mM SA was added to plates and the pH was raised to 8.0. The radial growth of mycelia for PH-1 and *Fshy1* mutant was measured and compared. Four biological replicates were performed. Comparisons between mean radial growth measurements were made using an ANOVA.

### Toxin Production in *Fgshy1*

*Fusarium graminearum* typically produces 15-ADON or 3-ADON in agmatine liquid culture. 15-ADON was measured in PH-1, *Fgshy1* mutants M1, M2, and M18 grown in liquid agmatine media (Gardiner et al., 2009). Cultures were grown in 20 mL of agmatine media for 7 days on a rotary shaker at 200 rpm in the dark at 28°C and then extracted with 8 mL of ethyl acetate. GC–MS analysis was performed on a Hewlett Packard 6890 gas chromatograph fitted with a HP-5MS column (30 m, 0.25 mm, 0.25 μm) and a 5973 mass detector.

### Gene Expression in Infected Wheat

For gene expression studies, wheat heads were inoculated with *F. graminearum* by dipping the whole wheat head into a 10^5^ conidia/ml suspension containing 0.02% Tween 20. Experiments were performed in triplicate. Wheat heads were collected at 3, 6, 24, and 48 h post-inoculation. Two heads were collected and combined for each time point. Three replicates were performed. Wheat heads were lyophilized and pulverized as described above. RNA was extracted from pulverized wheat head tissue using Trizol combined with column purification and on column digestion according to the manufacture’s instruction (Thermo Fisher Scientific, Waltham, MA, United States). cDNA synthesis, qPCR and gene expression analysis were performed as described above.

### FHB Pathogenesis Assay on Wheat

*Fusarium graminearum* PH-1 and *Fgshy1*-M1 and M2 mutant inocula were prepared from 4-day old mung bean cultures. The macroconidia were passed through a 45 μM strainer and adjusted to a concentration of 1 × 10^5^ conidia/mL for point inoculation. Twenty four heads from four pots were used for each strain. Wheat heads at flowing stage were inoculated with 10 μL of *F. graminearum* PH-1 or *Fgshy1* mutants. Inoculated wheat heads were covered with plastic bag for 3 days to keep high humidity. FHB disease development was scored at 7, 10, 14, 17, and 21 days post-inoculation (dpi). Disease severity was measured as a percentage of florets displaying symptoms. The mean percent disease for each treatment was compared independently for each time point using an ANOVA.

## Results

### Salicylate Hydroxylase Homolog Identified via *F. graminearum* Genome Mining

During the comparative analysis of genome sequences from 60 strains representing three North American *F. graminearum* populations, we identified a salicylate hydroxylase homolog (designated FgShyC, for salicylate hydroxylase candidate) unique to the NA2 (North American population 2) (Kelly and Ward, 2018). FgShyC contains 428 amino acids (aa), and shares the highest (81%) identity to a salicylate hydroxylase homolog from *F. proliferatum*, and 35% identity to the well-studied NahG from *P. putida* (You et al., 1991). Sequence comparisons and homolog search results showed that FgShyC is present only in NA2 strains, which are more aggressive than the NA1 population (Ward et al., 2008; Foroud et al., 2012). Therefore, we hypothesized that FgShyC provides the NA2 population a pathogenic advantage by degrading SA and suppressing the SA-mediated defense response of wheat.

### Ability of *F. graminearum* Strains to Degrade SA

**FIGURE 1:**
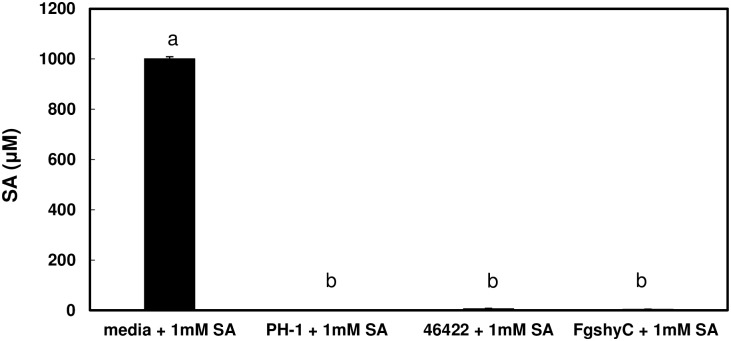
Salicylic acid (SA) degradation in *Fusarium graminearum* wild-type strains and *FgshyC* mutant. Total 5 × 10^4^ conidia were added to 4 mL mung bean liquid medium. Cultures were initially grown overnight in a shaker (200 rpm, 28°C). SA (1 mM, pH 8.0) was then added and growth was continued for 24 h. SA was extracted from whole cultures and concentrations were measured by GC/MS. Medium incubated without fungus served as a control. Bars represent the average of three replicates and SE. Letters above bars indicate significant differences (ANOVA, Tukey–Kramer HSD; *n* = 3; *P*<0.05). The experiment was repeated three times with similar results.

A previous study using high performance liquid chromatography (HPLC) analysis showed that *F. graminearum* strain DAOM180378 can metabolize SA (Qi et al., 2012). We examined the ability of *F. graminearum* strains PH-1 and NRRL 46422 to degrade SA in mung bean liquid medium. GC/MS analysis showed that only a trace of amount of SA was detected in PH-1 and 46422 cultures 24 h after addition of 1 mM SA, whereas the SA level was not altered in media controls without fungal inoculation (Figure 1). This demonstrated the ability of PH-1 and 46422 to degrade SA.

To examine whether FgShyC is involved in SA degradation, *FgshyC* of *F. graminearum* NRRL 46422 was disrupted using the split marker approach. The *FgshyC* mutant was identified by screening over sixty hygromycin resistant transformants by PCR amplification. The replacement of FgShyC was confirmed by genome sequencing and analysis. The ability of wild-type 46422 and the *FgshyC* mutant to metabolize SA was tested. When SA was added to liquid cultures of either wild-type 46422 or the *FgShyC* mutant it was degraded similarly (Figure 1). This demonstrates that *F. graminearum* produces salicylate hydroxylases and argues against this role for FgShyC. In addition, recombinant FgShyC protein expressed in *E. coli* (Figure 4A) did not convert SA to catechol in a colorimetric plate assay (not shown). Taken together, these results indicate that although FgShyC had sequence homology with salicylic hydroxylase genes, it did not function as a salicylate hydroxylase in our assays, and indicate that at least one other salicylate hydroxylase is active and responsible for the SA degradation activity observed in *F. graminearum.*

### *FgShy1* Is Induced by SA

**FIGURE 2:**
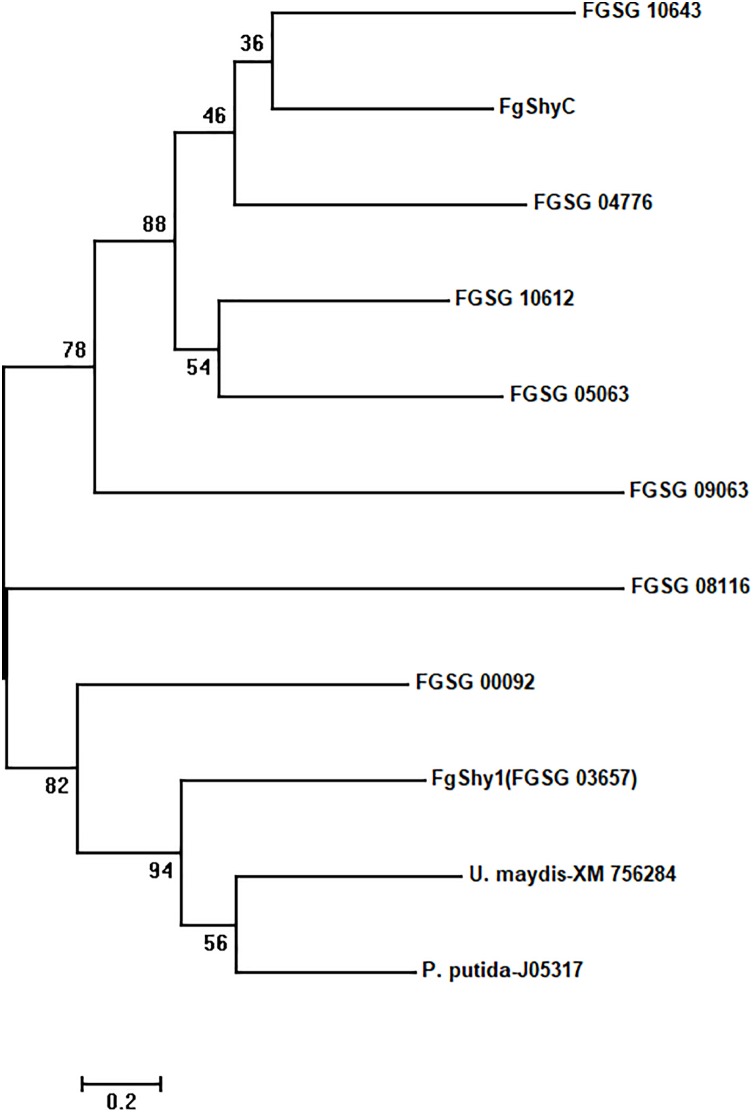
Phylogenetic tree showing the relationship between *Ustilago maydis* Shy1 (Accession No. XM_756284), *Pseudomonas putida* NahG (Accession No. J05317), FgShyC, FgShy1, and the other *F. graminearum* salicylate hydroxylase homologs. FgShyC from *F. graminearum* 46422, FgShy1 and the other eight salicylate hydroxylase homologs from the genome sequence of *F. graminearum* PH-1. These include: FGSG_03657 (FgShy1), FGSG_09063, FGSG_00092, FGSG_10612, FGSG_05063, FGSG_04776, FGSG_10643, and FGSG_08116. The MEGA 7 program was used to construct the tree using Maximum likelihood method with 1,000 bootstraps.

To identify additional salicylate hydroxylase candidates, we searched for FgShyC homologs in the *F. graminearum* PH1 genome by BLAST. Sequence examination and analysis identified eight salicylate hydroxylase homologs in the PH-1 genome. These eight candidates, which are also present in the NA2 strain 46422, share about 35% identity with each other and Shy1 from *U. maydis* and NahG from *P. putida* (You et al., 1991; Rabe et al., 2013). The salicylate hydroxylases from bacteria and fungi are flavin-dependent monooxygenases that contain the conserved FAD and NADH binding sites. These domains were identified in the eight salicylate hydroxylase homologs of *F. graminearum* except that FAD1 fingerprint 1 is absent in FGSG_10643 (Supplementary Figure S1). A phylogenetic analysis of these salicylate hydroxylase homologs was performed (Figure 2). FGSG_03657 (designated FgShy1) is most closely related to NahG from *P. putida* and Shy1 from *U. Maydis*, which degrades SA (You et al., 1991; Rabe et al., 2013).

**FIGURE 3:**
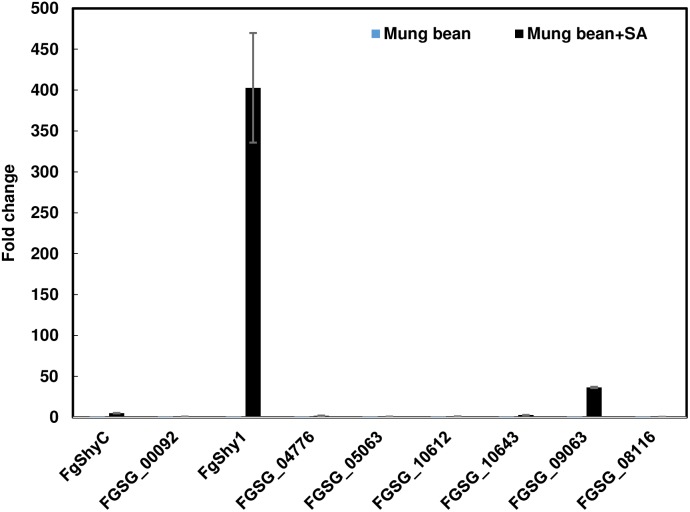
*FgShy1* induction in mung bean liquid medium with addition of 1 mM SA. *F. graminearum* strain 46422 was grown overnight and then incubated additional 2 h with or without 1 mM SA. Samples were collected for RNA isolation, cDNA synthesis and gene expression analysis. Fungal β-tubulin expression was measured and used as an internal control for transcript normalization. Gene expression levels for cultures grown in the presence of SA were determined relative to controls grown without SA.

Real-time PCR was used to determine if SA induces expression of any of the salicylate hydroxylase candidates. *F. graminearum* 46422 was grown in liquid cultures, with and without SA, and RNA was isolated. Comparative expression results revealed that one candidate, FgShy1, was highly induced by SA (Figure 3). FgShy1 transcripts were upregulated about 400-fold 2 h after addition of SA. FGSG_09063 was also induced, about 30-fold, whereas the other six salicylate hydroxylase homologs including FgShyC were not induced. These results demonstrate that FgShy1 is highly induced by SA and suggest that it may be an active salicylate hydroxylase.

### FgShy1 Is a Salicylate Hydroxylase

**FIGURE 4:**
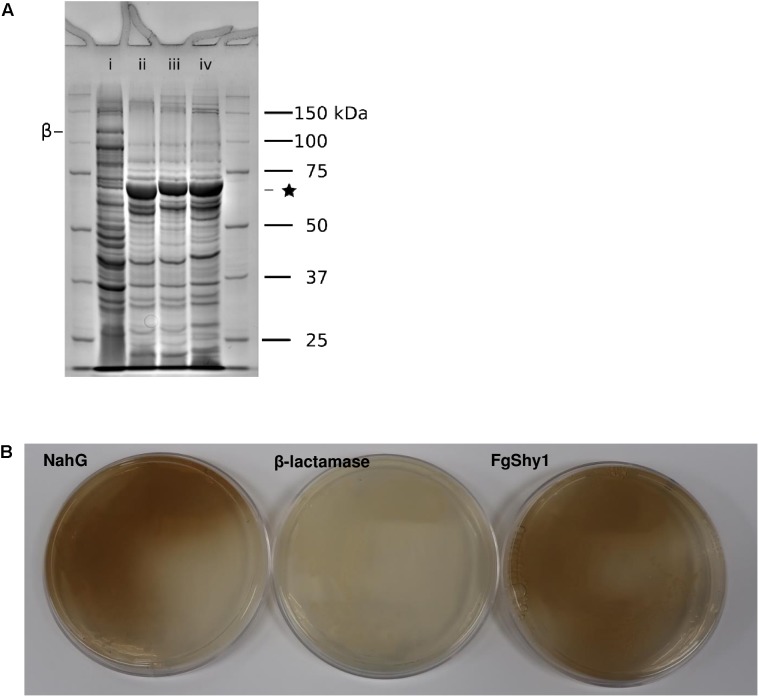
Overexpression of FgShyC and FgShy1 protein in *E. coli.* **(A)** SDS-PAGE analysis. (i) β-Lactamase control; (ii) NahG, (iii) FgShyC; (iv) FgShy1. ^∗^ Marks the salicylate hydroxylase fusion proteins. β Marks the β-lactamase protein. **(B)** Colorimetric plate assay of FgShy1 along with a positive control NahG and a negative control β-lactamase.

The salicylate hydroxylase activity of FgShy1 was confirmed by creating a heterologous strain of *E. coli* that produces recombinant FgShy1 from the encoding sequence and testing its ability to degrade SA using an *in vivo* plate assay. *P. putida* NahG (You et al., 1991) was used as a positive control. Both FgShy1 and NahG were expressed as fusions to Glutathione *S*-transferase as this was previously reported to improve NahG solubility (Rabe et al., 2013). Expression of both recombinant proteins was confirmed by inducing liquid cultures and analyzing their cellular protein content by SDS-PAGE (Figure 4A). Both strains produced large amounts of protein similar to the predicted sizes of 73.6 kDA (NahG) and 74.3 kDA (FgShy1) for the GST fusions. When grown on agar plates containing SA both the control NahG and FgShy1 producing strains turned the plates brown, as a result of auto-oxidation of catechol, and indicative of SA conversion to catechol (Figure 4B).

### *Fgshy1* Deletion Mutants Are Greatly Impaired but Not Disabled for SA Degradation

**FIGURE 5:**
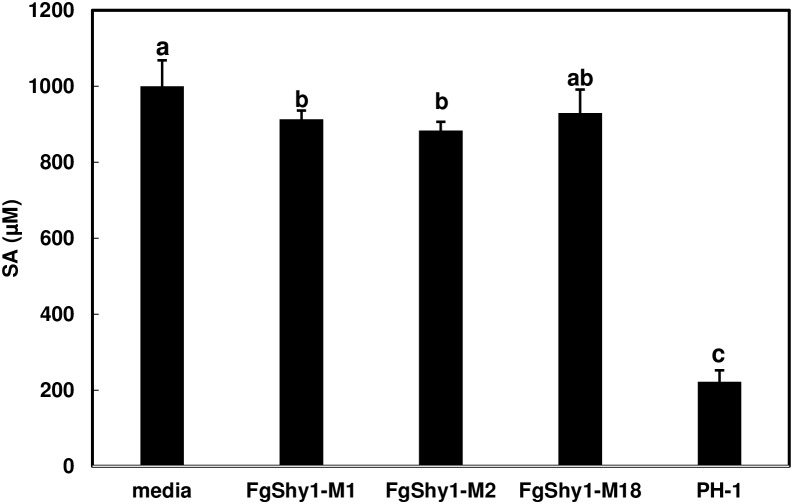
Salicylic acid degradation in *Fgshy1* mutants. Total 5 × 10^4^ conidia of PH1 or *Fgshy1* mutants were added to 4 ml mung bean liquid medium. Cultures were grown overnight in a shake incubator. Each culture was then supplemented with 1 mM SA (pH 8.0) and grown for 24 h. SA was extracted from whole culture and analyzed with GC/MS. Bars represent the average of three replicates and SE. Letters indicate significant by Dunnett’s method (*n* = 3; *P* < 0.05).

To determine if FgShy1 is essential for SA degradation in *F. graminearum*, deletion mutants were created by replacing FgShy1 with a hygromycin-resistance gene (Hyg). A total of 19 hygromycin resistant transformants were obtained. PCR amplification indicated that three of the transformants lacked the *FgShy1* coding region (Supplementary Figure S2). All three *Fgshy1* mutants (M1, M2, and M18) grew the same as wild-type on plates and in liquid media. *FgShy1*-M1, M2, and M18 were examined for their ability to degrade SA in mung bean culture. GC/MS analysis showed that the wild-type strain PH-1 degraded approximately 80% of the added SA whereas *Fgshy1* deletion mutants were significantly reduced in their ability to degrade SA in liquid culture. Compared to media control, *Fgshy1*-M1 and M2 mutants were able to degrade 10 and 11% of the SA respectively but *Fgshy1*-M18 did not significantly reduced the amount SA added (Figure 5). Therefore, Fgshy1 is the key active enzyme involved in the observed SA degradation by *F. graminearum*, but there may be at least one other protein that modestly contributes to the activity found in the wild-type strain.

### Effect of SA on *Fgshy1* Mutant Growth

**FIGURE 6:**
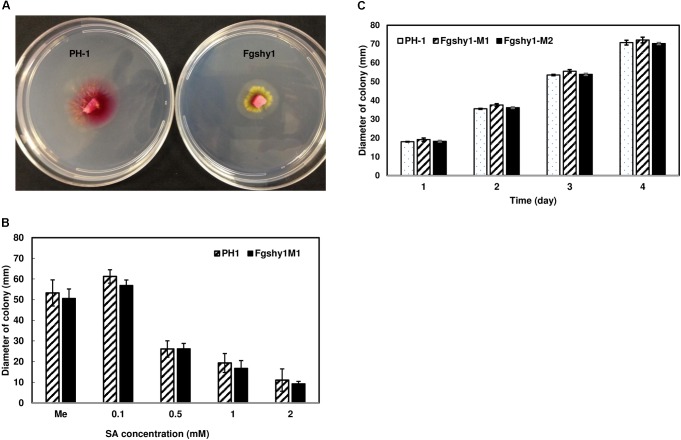
*Fgshy1* growth on agar containing SA. **(A)** The yellow hyphae of the *Fgshy1* mutant with addition of 1 mM SA under acidic condition. **(B)** Growth of *Fgshy1* on 1% agar plates under acidic condition with different SA concentrations. **(C)** Growth of *Fgshy1* with 10 mM SA under neutral conditions. Wild-type PH-1 served as control. Values are averages from four biological replicates. Me, methanol. Means at each different concentrations **(B)** and time points **(C)** were analyzed independently using an ANOVA, no significant differences were detected (*P* > 0.05).

Previous work demonstrated that *F. graminearum* growth is inhibited by SA under acidic growth conditions whereas *F. graminearum* can use SA as a carbon source under basic conditions (Qi et al., 2012). We therefore examined whether or not SA affects growth of the *Fgshy1* mutant differently than the wild-type PH-1 strain under acidic and neutral conditions. As expected, with an increase in SA concentration, the colony growth of both *Fgshy1* and PH-1 was significantly reduced under acidic conditions. With addition of SA at concentrations of 1 and 2 mM, the *Fgshy1* mutant grew slightly slower than the wild-type PH-1 and the hyphae turned yellow (Figure 6A). However, no significant growth difference between *Fgshy1* mutant and PH-1 was found by statistical analysis (Figure 6B). Additionally, growth of the *Fgshy1* mutant and PH-1 were not inhibited when grown on basic agar media (pH 8.0) containing 10 mM SA (Figure 6C). Our results suggest that FgShy1 is not essential for the growth of *F. graminearum* on agar medium with SA, suggesting additional enzymes or other SA degradation pathways exist.

### Induction of *FgShy1* and Its Homologs During Wheat Infection

**FIGURE 7:**
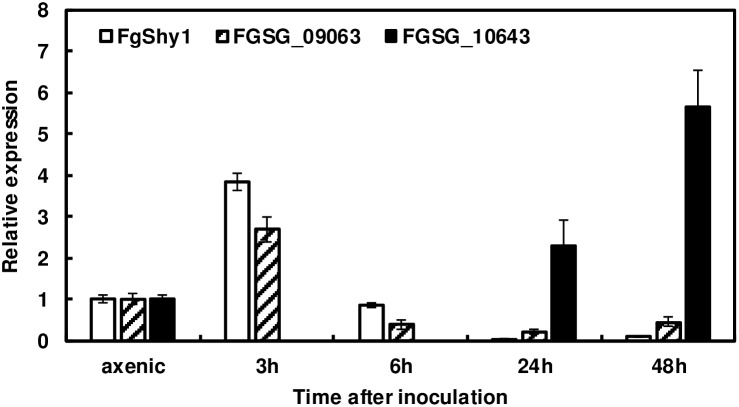
The expression of *FgShy1* and its homologs in infected wheat. RNA was isolated and cDNA were prepared from wheat heads collected 3, 6, 24, and 48 h after dip inoculation. Six heads were collected at each time point and two heads were combined as one sample for RNA isolation and cDNA synthesis. Gene expression was determined by RT-qPCR. Fungal β-tubulin expression was used for transcript normalization. Fold changes of gene expression were relative to *F. graminearum* 46422 axenic cultures grown on V8 agar for 7 days.

To determine whether FgShy1 and its homologs are induced during wheat head infection, we examined their transcript levels at various time points after dip inoculation with *F. graminearum* 46422. *FgShy1* expression was induced about fourfold at 3 hpi Similarly, FGSG_09063 was induced about threefold at 3 hpi and FGSG_10643 was upregulated about 2- and 6-fold at 24 and 48 hpi respectively (Figure 7). The remaining six FgShy1 homologs did not show signs of induction (data not shown). Induction of *FgShy1*, FGSG_09063, and FGSG_10643 in infected wheat heads suggests they may play a role in FHB development.

### Disruption of *FgShy1* Does Not Significantly Affect *F. graminearum* Virulence and DON Production

**FIGURE 8:**
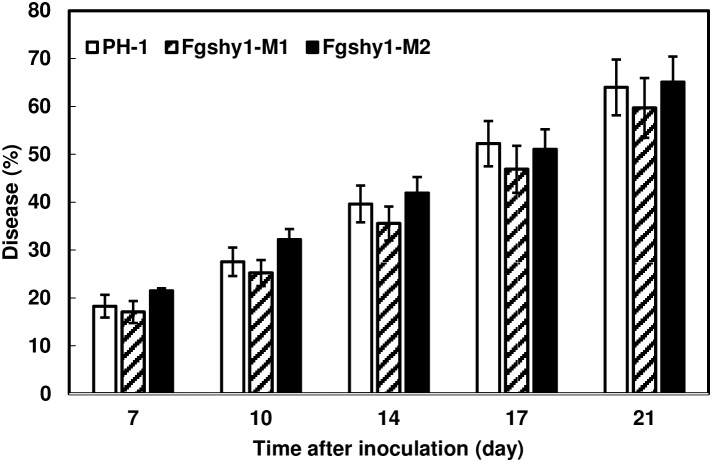
Fusarium head blight (FHB) assays in wheat. Point inoculation (10 μl spore suspension containing 1,000 conidia) was performed on wheat florets (cv. Norm) with *F. graminearum* wild-type PH-1 and *Fgshy1* mutants M1 and M2. FHB disease progression is expressed as the percentage of inoculated florets exhibiting disease symptoms at 7, 10, 14, 17, and 21 days post-inoculation (dpi). Comparisons between the mean percent disease of each treatments were made for each time point independently using an ANOVA, no significant differences were detected (*P* > 0.05).

A previous study showed that DON production in *F. graminearum* is reduced by addition of SA (Qi et al., 2012). We measured DON production in *Fgshy1* mutant in agmatine liquid medium. Our results demonstrated that 15-ADON production was similar in the parent strain PH-1 and the *Fgshy1* deletion mutants (not shown). However, the induction of *FgShy1* in infected wheat raises the possibility that FgShy1 degrades plant-produced SA and suppresses SA-mediated defense response. To test if disruption of *FgShy1* affect FHB development, we performed virulence assays on wheat heads using *Fgshy1* mutants M1 and M2. Head blight development was assessed following point inoculation with *Fgshy1* mutants and the wild-type strain PH-1 over a 21-day period. There was no significant difference between the wheat heads inoculated with *Fgshy1* mutants and those inoculated with PH-1 (Figure 8). This suggests that disruption of *FgShy1* alone has no significant effect on *F. graminearum* pathogenesis.

## Discussion

Here we identify and demonstrate functionality of FgShy1, a salicylate hydroxylase, encoded by *F. graminearum*. *FgShy1* is highly induced by addition of exogenous SA. Heterologous expression of FgShy1 resulted in converting SA to catechol. The *Fgshy1* deletion mutants are greatly impaired in the ability to degrade SA in culture, suggesting that FgShy1 is the key active enzyme for SA degradation in culture. Fgshy1 mutants did not affect FHB pathogenicity, we cannot rule out that additional SA-degrading enzymes are produced during *F. graminearum* and plant interaction.

We identified eight salicylate hydroxylase homologs in NA1 strain PH-1 and nine in NA2 strain NRRL 46422. Salicylate hydroxylases belong to the flavin-dependent monooxygenase family, which catalyzes a variety of oxygenation reactions on specific substrates (Huijbers et al., 2014). Since only *FgShy1* was highly induced by SA in mung bean medium, we examined its SA-degrading activity by heterologous expression in *E. coli* and gene deletion mutagenesis in *F. graminearum*. The *E. coli* strain expressing *FgShy1* degraded SA to catechol, as previously shown for a salicylate hydroxylase from the fungal endophyte *E. festucae* (Ambrose et al., 2015). Although the *Fgshy1* mutant are greatly impaired in the ability to degrade SA, these mutants still can degrade SA in liquid medium. These results suggest that additional salicylate hydroxylases or degradation pathways exist in *F. graminearum*. The existence of multiple salicylate hydroxylases in *F. graminearum* may suggest that they play an important role during *F. graminearum* wheat interaction. Only one salicylate hydroxylase homolog was identified in *Liberibacter* bacterial species including *L. asiaticus*, *L. africanus*, *L. solanacearum*, and *L. americanus* (Li et al., 2017). Three salicylate hydroxylase homologs are present in *U. maydis* but only Shy1 has been demonstrated to have SA degrading ability (Rabe et al., 2013). Because of the relatively low sequence identity between salicylate hydroxylases it is difficult to identify them solely on the basis of sequence data. In our experiments, FGSG_09063 expression was slightly induced. In the microarray analysis, both *FgShy1* and FGSG_09063 were upregulated with SA treatment (Qi et al., 2012). FGSG_10643 was not induced by SA in mung bean liquid medium, but it was induced during wheat infection. In addition FGSG_10643 lacks FAD1 fingerprint 1 (Supplementary Figure S1). Further efforts are underway to determine whether FGSG_09063 and FGSG_10643 are salicylate hydroxylases. Induction of *FgShy1* and FGSG_09063 by SA suggests *F. graminearum* can sense SA. An SA sensing regulator, Rss1, was recently identified in *U. maydis* (Rabe et al., 2016). However, Rss1 homologs are not present in *F. graminearum*, indicating *F. graminearum* has a different SA sensing and regulatory system. Further investigation is needed to determine the molecular mechanism of *F. graminearum* sensing and regulation of SA degradation.

FgShy1 overexpressed in *E. coli* converted SA to catechol. This suggests that *F. graminearum* can utilize SA as a carbon source. The degradation pathway of SA to catechol is present in *Fusarium* sp. strain BI, which can utilize SA as carbon source to support its growth (Dodge and Wackett, 2005). In fact, the ability to degrade SA is a common phenomenon among fungi. Five genera of fungi, *Aspergillus, Trichosporon*, *Trichoderma, Glomerella*, and *Rhodotorula*, have been shown to metabolize SA (Wright, 1993). The mung bean liquid medium used in this study is nutrient deficient, and *FgShy1* is highly induced by addition of SA in mung bean medium. This suggests that FgShy1 is associated with utilization of SA as a carbon source under nutrient deficient conditions. Unlike other fungi such as *U. maydis*, *F. graminearum* can grow on agar plates without additional sugars. It is difficult to determine whether *F. graminearum* can utilize SA as a sole carbon source. Although no significant growth inhibition was observed in *Fgshy1* mutant, we observed that hyphae of *fgshy1* mutant turned yellow on plates containing SA. The mechanism for this phenomenon is not clear. In addition, FgShy1 shares strong identity to hypothetical proteins in *F. pseudograminearum* (99%), *F. poae* (93%), and *F. oxysporum* (84%). It will be interesting to determine if these salicylate hydroxylase homologs degrade SA.

*FgShy1* is induced during wheat infection indicating that FgShy1 may degrade host SA and suppress SA-mediated plant defense. However, FHB severity was not altered significantly in wheat heads inoculated with the *Fgshy1* mutant compared with wild-type control. Since our results showed that the Fgshy1 mutants are greatly impaired but still have the ability to degrade SA in mung bean liquid culture (Figure 5), we suspect that at least one additional salicylate hydroxylase is present in *F. graminearum*, which may compensate for the loss of FgShy1 function and enable degradation of SA in the *Fgshy1* mutant. Although the role of SA-mediated signaling has been demonstrated in the *Arabidopsis*–*F. graminearum* interaction (Makandar et al., 2010), some studies in wheat have shown that SA contributes to FHB basal defense (Makandar et al., 2010), whereas other studies showed treatment with SA or SA functional analogs did not affect FHB development (Yu and Muehlbauer, 2001; Li and Yen, 2008). These studies suggest that the orderly coordinated and activation of hormone pathways are critical for plant resistance (Ding et al., 2011). It is also possible that *F. graminearum* uses SA as a carbon source during its necrotrophic stage as other microorganisms do (Hadibarata et al., 2012). Therefore, characterization of additional salicylate hydroxylases and generation of mutants lacking all salicylate hydroxylases will be necessary to clarify whether salicylate hydroxylases are virulence factor during FHB pathogenesis.

Collectively, the results of our study demonstrate that *F. graminearum* FgShy1 is an active salicylate hydroxylase. Disruption of FgShy1 significantly reduces SA degradation ability in culture. Mutation of FgShy1 did not alter FHB development in our assays, which indicates that FgShy1 is not essential for FHB. However, we cannot excluded that FgShy1 detection may play a subtle role due to redundancy of salicylate hydroxylases. Two bacterial salicylate hydroxylases have been found that suppress plant immunity via SA degradation (Lowe-Power et al., 2016; Li et al., 2017). Although the fungal salicylate hydroxylase shy1 from the biotrophic pathogen *U*. *maydis* is not associated with virulence of the pathogen (Rabe et al., 2013), the coordinated expression of horomones has been associated with plant defense against *F. graminearum* (Ding et al., 2011). To our knowledge, a direct connection between salicylate hydroxylases and fungal pathogenesis has not been demonstrated. Characterization of additional *F. graminearum* SA hydroxylase is underway, which will shed light on the direct role of SA during FHB pathogenesis.

## Author Contributions

GH conceived and designed the experiments. GH, TU, TN, MV, and SM performed the experiments. GH, TN, MV, and SC analyzed the data. GH, AK, and TW contributed reagents, materials, and analysis tools. GH, TN, and MV wrote the manuscript.

## Conflict of Interest Statement

The authors declare that the research was conducted in the absence of any commercial or financial relationships that could be construed as a potential conflict of interest.

## Supplementary Material

FIGURE S1 Comparison of deduced salicylate hydroxylase amino acid sequences. FgShyC from *Fusarium graminearum* 46422, FgShy1 and the other eight salicylate hydroxylase homologs from the genome sequence of *F. graminearum* PH-1. *Ustilago maydis* (Accession No. XM_756284), and *Pseudomonas putida* (Accession No. J05317). The conserved FAD1 fingerprint 1 and 2, substrate active sites, NADH-binding domain and FAD domains are colored.

FIGURE S2 Polymerase chain reaction screening of *fgshy1* mutants. M, DNA Marker; Lanes 1–10: transformants; Lane 11: PH-1; Lane 12: water control. Transformants 1 and 2 do not show target gene amplification, indicating deletion of *FgShy1*.

TABLE S1 Primers used in this study.
