# Supplementary material for: Characterization of a Fusarium graminearum Salicylate Hydroxylase
Source: Front Microbiol. 2019 Jan 8;9:3219. doi: 10.3389/fmicb.2018.03219 (PMC6331432; doi:10.3389/fmicb.2018.03219)
Supplement: FIGURE S1 — Comparison of deduced salicylate hydroxylase amino acid sequences. FgShyC from Fusarium graminearum 46422, FgShy1 and the other eight salicylate hydroxylase homologs from the genome sequence of F. graminearum PH-1. Ustilago maydis (Accession No. XM_756284), and Pseudomonas putida (Accession No. J05317). The conserved FAD1 fingerprint 1 and 2, substrate active sites, NADH-binding domain and FAD domains are colored. [file Image_1.PDF]

```
P.pudita        -----------------MKNNKLGLRIGIVGGGISGVALALELCRYSHIQ-VQLFEAAPA 42
U.maydis        -------MSSTSTSTSTGTKATKDFSVAIIGGGIGGLTLAIGLHE-RGVP-IHVYESASK 51
FgShyC          ------------------------MQVVIVGAGLGGIACGIACRR-QGLD-VTILERAPE 34
FgShy1          --------------MTQTSSKQKAFDIAIVGGGIAGLTLAIALHR-RNIP-VTLFERADN 44
FGSG_09063      -----------MIKDTPSMPNWKHLSVAVVGGGIGGMSVAIALRR-AGHS-VTIYERSDF 47
FGSG_00092      ----------------MAVTSEKEFHVAIVGAGIGGLALAMALHK-KGIS-FTLYEDAKE 42
FGSG_04776      MLERLHRSFQHVNGETSHTKTQVELRIVVVGAGLGGLATAIALAR-RGHK-VVVLEQAAA 58
FGSG_05063      ------------------MADIKDLHIAIMGAGMGGLGAALALAK-RGFKHIDVYETASN 41
FGSG_08116      ---------------------MSSPKVAIIGAGLSGLALALALHQ-QGIE-STAYEQQSA 37
FGSG_10612      MDTLRDRWV------PCSQRAARPLNVIIVGAGIGGLTAGISLSQ-TGHS-VTILERVNK 52
FGSG_10643      ------------------------------------------------MS-IQLTSSNVV 11
                                                                       .
                                      FAD1 fingerprint 1

P.pudita        FGEVGAGVSFGPNAVRAIVG--LGLGEAYLQVADRTS-EPWEDVWFEWRRGSDAS----- 94
U.maydis        FSEIGAGIAIGPNSQAALER--LGLYESFVQFAD----FPSRNLFFQWRLAEPEEQ---- 101
FgShyC          LSEIGAGIQVPPNACRALDY--IGVLDKIKGKAT----EILVRHLRRWDNGDILAT---R 85
FgShy1          FHEIGAGVSFTPNAVQAMKVCHPGVSEAFYKVCTWNSWESKKKTWFDFLDGTTEDG---- 100
FGSG_09063      AGEVGASVSCAANGTRWLHE--WDVDVAKGDPVV-----LKKLINRDWKTGEPVSV---- 96
FGSG_00092      FSAVGAGIGFAPNGMRTMDLIEPGFRPLYEKICVGNKGDDAQTIFFEGMLLEEGFGRDQP 102
FGSG_04776      LGEVGAGIQIPSNSARLLLR--WGIGPYLEQYAV----KPESMTFRRWENGDPIGY---T 109
FGSG_05063      LGFVGAGIQMPPNVGRILDR--LGCWNDIEKEAT----RVAGSSIRQGSTNEELAH---- 91
FGSG_08116      PLDIGGAIMLSPNSLRALDK--LGVFQRMLPRSY-----KFNELYFLSQDDKLVD----- 85
FGSG_10612      IDEVGAGIQLAPNASRILNR--LGVLEEIMEHAT----VLERVSIRRYSGDDELST---V 103
FGSG_10643      FLKVGAGLQVTPNASRLLHQ--WDLPQRFWDSVA----EPTYLAVHRYSGQLLALE---E 62
                   :*..:    *    :     .
                  Substrate active site

P.pudita        --YLGATIAPGVGQSSVHRADFIDALVTHLPEG-------IAQFGKRATQVEQ---QGGE 142
U.maydis        -TLLSETICKKYGMASIHRAELLDTFIKRVPSH-------VCSFGKRLQSLQQPTTADGK 153
FgShyC          KYGSVSYEKYGYPAMVIHRADLQRTLIETAEELGA-----QIRTGCDVSKADF---EATE 137
FgShy1          --KTAFSIKTSLGQNGVHRAHFLDELIHLLPSE-------RVQFGKQIEQAEE--DADGK 149
FGSG_09063      YDLDDYEKRWGFVYNMFHRQYMHAMLKDTAMGEDGEGEPVKLLVKHKCTSIDI---PNG- 152
FGSG_00092      WVGKSGWGHPDYIRKSAHRKELLDIMTSFVPKD-------SVKFSKRLTKIKQ---SPDG 152
FGSG_04776      RLSPDFEITYGAPYFVIHRADFHRALCRLAEDLGV-----TIVTDSKVVEYDE---AAPS 161
FGSG_05063      VDMPDLKGQYGYSHLCGHRSSLAGHMYEACKKQDS----IKFHFSTSLVEVET---FSPK 144
FGSG_08116      VFEFGNEEKYGYTGIRVYRFELINVLLDLIREAG-----IKVQYGRKFDQIVN--ETEQS 138
FGSG_10612      PLMPGTGLKYGAPLSVIHRGDLQETLLNAARKAGC-----QILTSQTVICADP---EFSA 155
FGSG_10643      DFDKKIRKQYGAPFVDVHRVDLQLSLLERAQELGA-----KLKLSQKVIDIDF---NTPK 114
                                 :*  :   :

P.pudita        V----QVLFTDGTEYRCDLLIGADGIKSALRSHVLEGQGLA-----PQVPRFSGTCAYRG 193
U.maydis        V----KMTFHDGSTHEADLVIGCDGIHSRVRGALDPNTAGPSAVAGSDALVWSGTWAYRG 209
FgShyC          VV------LSNGEHVKADVILGADGIWSTLRSQVV---GQ------SIEPTETGDLAYRG 182
FgShy1          I----RMTFSDGTTAYADALIGCDGIGSRVRKIIVGENHP------SARPQYSHKYAYRG 199
FGSG_09063      -----TITFDNGVTVKHDLIVGADGIGSVVRGILGIHP----------EKKPSDQSCLHC 197
FGSG_00092      V----TLSFQDGTTANCDILAGADGIKSTVRGHVLEKHPN------QIAPVYAGAYCYRA 202
FGSG_04776      AS------TSDGREYSADLVIAADGVKSIARSVVLG--GP------DLPAQRTGFAAYRA 207
FGSG_05063      VSFKLKPRDGEPYTSQADILLGADGIKSVTRSQILQQVDA------TPEEAETGQAAYRI 198
FGSG_08116      V----TWRFTDGSEETADLLIGADGIHSRVRSYLYPDL----------TPKFTNMIGVTA 184
FGSG_10612      SVQVRDNQTREMSWLHGDLLIAADGIKSTIRQQMALTDGF------NDGPVCTGSAAYRL 209
FGSG_10643      IT------TQDGTEAKADLIIAADGLWSRCRSAFL---GT------KDMPKPTGDLAYRV 159
                          :        * :  ..**: *  * .                 :
                                 FAD and NADH binding

P.pudita        MVDSLHLREAYRAHGIDE--HLVDVPQMYLGLDG---HIL-TFPVRNGGIIN-----VVA 242
U.maydis        LIPRQEFVAALG-KDKGE--FYADTAQMMLAKDS---HIL-IFPIQGGKTVN-----IVA 257
FgShyC          TFTRKQLEELNDPEVLRFCEENKQTLTLWLGPLK---HAV-FYAIRGGELWN-----LVL 233
FgShy1          LIPMDKAIEAVG-----E--ERARNACMHMGPDG---HIL-TFQVNHGEKLN-----IVA 243
FGSG_09063      NVTTEEAVKAG---LVDY--SQNSALEYWGGQEGKWDKIV-LSPCNGGKLLS-----YYC 246
FGSG_00092      VIPMDEAYEILG--------DLTDVAKFYFGHKK---SAI-SYRITQGNEFN-----YLL 245
FGSG_04776      TVDTEDMKCDKDTSWL----LEKPGINIWIGEDR---HVM-TYCIAGGNSFN-----LVL 254
FGSG_05063      MLNREDMA--HDPELLAL--IDSDEVVRWVGEKR---HII-AYSIADKSIYN-----LST 245
FGSG_08116      AVPTSQLKLEEG--------GYKLPATFMHDKRG---AFVIAPQLADGSEVLIGKQKVFV 233
FGSG_10612      LVPIDKIK--QDPLLSGM--LKQNVAMRYMGPGG---HIM-AYPLKNNTLYN-----LVL 256
FGSG_10643      VLNLDDIK---DPELIDW--VKHCSCHFWIGPGA---HAV-GYSLRGGNMYN-----IVL 205
```

```
                 .   .                               :

P.pudita        FISD-RS--EPKPTWPADAPWVREASQREMLDAFAGWGDAARALLECIP--APTLWALHD 297
U.maydis        FKTD-RTRWPERTPFRKGEPWIQETSQEALLDDFATYSSDLIKMLKCIE--KPNKWALHQ 314
FgShyC          LTPDKMKKG----QRT------EKGDLGEMCQEFEGWDPILTKITTCFS--STMKWKLCH 281
FgShy1          FRTD-PNEWDNPSKMT------KTARRQDALDDFKGYNSLVRNLLALTDE-TLSVWAIFD 295
FGSG_09063      FFPREQGDYTTQAWGS------DSLPVEDLLKPYPQLDSQVLGHLAIGK--EIQPWRLWV 298
FGSG_00092      CVADGLNAWKIPHAVT------ELIPHEQMMADFEGPGIDPKFRELLAKA-KPVKWGFFH 298
FGSG_04776      SHVDHSSPS----TWN------SETAIQDMQDSFRDWDPKLQKVIMMIK--KTIKWPLMT 302
FGSG_05063      AHPDENFAGAPSITYT------TKGSKEVMLKVFDTFCPLVQKMLNLVPEGEVCEWRLRM 299
FGSG_08116      GEDPGRDAWK---AMNSDKTWCVDFLKEGK-EDYP--AIVSNATSHISPE-KVNLWPFYL 286
FGSG_10612      VHPVKRCNLE--DVWT------SKGDRQEMLDFYQNWSPAIRRWLELADQ-DVMEWNLYS 307
FGSG_10643      LVPDDLPPG----ASR------LPGSVDQMKALFEGWDPILLRFLDLVT--EVDRWKLMH 253
                                                 :                     * :

P.pudita        L----AELPGYVHGR--VVLIGDAAHAMLPHQGAGAGQGLEDAYFLARLLGDTQAD---- 347
U.maydis        VV---PSLSSYVNGR--VIVSGDAAHGGVPHQGAMAGQAIEDALFLSKLLSHPKVN---- 365
FgShyC          H----GELDTWVKQA--FALLGDSAHPTLPYQSQGAAMAFNDAAVIGALLGRFHQIDKGQ 335
FgShy1          TG--DNPVPTFYKGR--IAILGDAAHATSPHHGAGAGFCIEDSAVMAELLADERVQS--- 348
FGSG_09063      H----QPYPYIHKGN--VCLLGDAGHPMMPHQSQGACMAIEDAAALGILFSKRYFN---- 348
FGSG_00092      H----RHTAAYFRDR--VALVGDSAHASLPFQAAGAAQGLEDALVLSALVAELAKQHQR- 351
FGSG_04776      G----SRLQTWISRSQKLVILGDAAHAMVPYMSQGAAMAVEDGAALAAAISEVTYKE--- 355
FGSG_05063      Y----KPLPTWTHGA--VALLGDACHPTLPHLSQGAAMAIEDGSTIAEVLCLAPDTK--- 350
FGSG_08116      LPKLDKWASSDKHGR--VAILGDAAHAIPPTAGQGVNQAFEDVYTFAGVLGQLKQSN--- 341
FGSG_10612      Y----RPLPQWVKGS--TALIGDACHPMLPFVAQGAANAIEDAAVLATALTCTAD----- 356
FGSG_10643      H----DEMQHWINDQSNFVFIGDACHPMLPYLAQGANSAIEDGAVLGLLLGAVEARN--- 306
                                   . **: *   *  .  .   .:*   :.  .
                                        FAD1 fingerprint 2
P.pudita        ----AGNLAELLEAYDDLRRPRACRVQQTSWETGELYELRDPV-VGANEQLL--GENL-- 398
U.maydis        ----NANLTRALQVYDKIRMPRGNKVVETSLEAGDTYEFRGV--AADDPHKL--GQHL-- 415
FgShyC          LEAKGMTLHKVLKIFEAGQKPYSSLNVQGAVKNRVMYHLPDGKAQQQRDMEFSQMMAE-- 393
FgShy1          ----RSDLEKVFAAYDESRRERTQWLVQSSRFVGDAYEWRAKG-VGKDIPGI--EREI-- 399
FGSG_09063      -----GDIAQSLSVYEKVRLPRATRVQAAAAKAAYNINERIGFSVNKDVSTYKVENEK-- 401
FGSG_00092      GASQQPAIQAALTAYDSVRRPRAQKQLEQAAEVGDMMFFQHKE-AGDDMLKI--LPHL-- 406
FGSG_04776      ------EVPMALRVFEKERMQRSYGMQSASLVNGRLWHFPDGPLQQARDLGMRAEVEERS 409
FGSG_05063      ----PETIAKCLKVYEQSRKEWTSSLVEMAYLSGRTLHLGEGKAKEERDRMFKEHKLS-- 404
FGSG_08116      ----GKGLSDTLDRWQKGRQERVDKIIELNNEINKRRMPKVA--------GV--EVET-- 385
FGSG_10612      -------VKLALKMYEVIRKDRGEQIAASAAKTAHTLHLPDGPEQRDRDEAIVS---G-- 404
FGSG_10643      ------QIPEALKLYEKMRKSRGEAIVRETFKQRESFHMVDGPQQVKRDEIFLSQLGKKE 360
                       :   :  ::  :

P.pudita        -----------ATRFDWLWNHDLDTDLAEARARLGWEHGGG--GALRQG----------- 434
U.maydis        -----------VERFDHIWDYDLDAENDQMNQ---WIQQNL------------------- 442
FgShyC          --SRSDWTWIDGQYQKRIIGTDLVKVALEKFDSELNK----------------------- 428
FgShy1          -----------NERIGVISNVEIAKSCEMARELLA------------------------- 423
FGSG_09063      ----------EVLTIEEMNTYDMYKDIEEKLAKAKGDKYDGFINGLPVGLVLPNGVTIGT 451
FGSG_00092      ----------QQGRFNWLWFHDMNDDAKEAVSRMHKLIGSN--PPSRI------------ 442
FGSG_04776      -FVESTNQWSDPVTQLWAYGYDAEQAIENLWRTERASVES-------------------- 448
FGSG_05063      --GSVPDKWTSPDVQKKIYSNDCVAKVRDEFETLFAAV---------------------- 440
FGSG_08116      ----------KPFDVDWLYSVDLNEAVKQFVGSN-------------------------- 409
FGSG_10612      --TQNPDRWSNGQWQDYMYGVDVMKATVDLWNVMQERTVV-------------------- 442
FGSG_10643      LKAPFPSRWTCPEVQPWLYGYDAYKEVHDALAKNSFTRFSVVAQPA--------SPVVTS 412
                                     :

P.pudita        -------------     434
U.maydis        -------------     442
FgShyC          -------------     428
FgShy1          -------------     423
FGSG_09063      -------------     451
FGSG_00092      -------------     442
FGSG_04776      RL-----------     450
FGSG_05063      -------------     440
FGSG_08116      -------------     409
```

FGSG_10612	RSRQVNL------	449
FGSG_10643	RSWLSRLLTYIHS	425
