# Supplementary figures and images for: Characterization of a Fusarium graminearum Salicylate Hydroxylase
Source: Front Microbiol. 2019 Jan 8;9:3219. doi: 10.3389/fmicb.2018.03219 (PMC6331432; doi:10.3389/fmicb.2018.03219)

## Slide 1
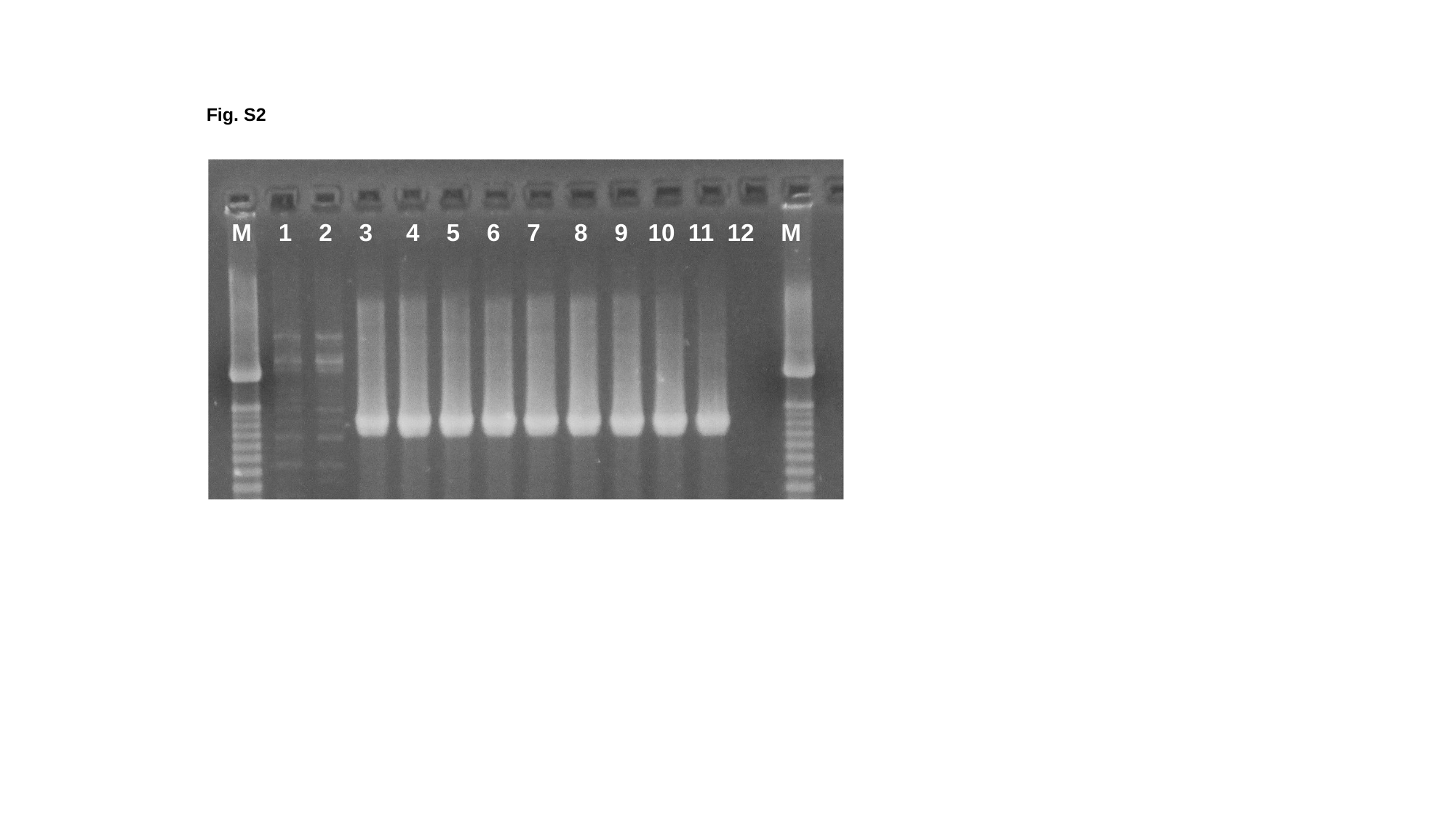

Fig. S2
M 1 2 3 4 5 6 7 8 9 10 11 12 M

Supplement: FIGURE S2 — Polymerase chain reaction screening of fgshy1 mutants. M, DNA Marker; Lanes 1–10: transformants; Lane 11: PH-1; Lane 12: water control. Transformants 1 and 2 do not show target gene amplification, indicating deletion of FgShy1. [file Presentation_1.PPTX]
