# Supplementary material for: Characterization of a Fusarium graminearum Salicylate Hydroxylase
Source: Front Microbiol. 2019 Jan 8;9:3219. doi: 10.3389/fmicb.2018.03219 (PMC6331432; doi:10.3389/fmicb.2018.03219)
Supplement: TABLE S1 — Primers used in this study. [file Data_Sheet_1.docx]

**Table S1 Primers used in this study.**

|  |  | |
| --- | --- | --- |
| **Gene Name** | | **Primer sequence** |
|  | |  |
| **RT-PCR** | |  |
|  | |  |
| FGSG_10612-For | | GTACGGAGTGGATGTGATGAAG |
| FGSG_10612-Rev | | CTACAGGTTTACTTGCCGAGAC |
|  | |  |
| FGSG_00092-For | | GCGTGGCAGATGGTCTAAAT |
| FGSG_00092-Rev | | GATTCCAGGGCCTTCAAAGT |
|  | |  |
| FGSG_05063-For | | GCCAAGTGTCTCAAGGTGTAT |
| FGSG_05063-Rev | | CCTTCACCGAGATGTAGTGTTC |
|  | |  |
| FgShyC-For | | TTCAAGGAGCGGTCAAGAATAG |
| FgShyC-Rev | | CTTCGAGATTCCGCCATCAT |
|  | |  |
| FGSG_08116-For | | TCTTATTCGTGAAGCGGGTATC |
| FGSG_08116-Rev | | ATCAGTGAAGCGCCAAGTAA |
|  | |  |
| FGSG_04776-For | | GAGGACATGAAATGCGACAAAG |
| FGSG_04776-Rev | | CCAGCGATGCAGTAAGTCATA |
|  | |  |
| FgShy1_For | | CAAGACTGCTTTCAGCATCAAG |
| FgShy1_Rev | | CTGACGGTAGCAGATGGATAAG |
|  | |  |
| FGSG_10643-For | | GTTGAGGCTCGTAACCAAATTC |
| FGSG_10643-Rev | | CCCGCTGCTTAAATGTTTCTC |
|  | |  |
| FGSG_09063-For | | CGAGAATGAGAAGGAGGTCTTG |
| FGSG_09063-Rev | | ACTTATCACCCTTCGCCTTG |
|  | |  |
| FGSG_09530-β-tubulin-F | | TCCAGGGTTTCCAAATCACC |
| FGSG_09530-β-tubulin-R | | GGAACGACGGAGAAAGTTGC |
|  | |  |
| **Mutagenesis** | |  |
|  | |  |
| OSC-F | | CTAGAGGCGCGCCGATATCCT |
| OSC-R | | CGCCAATATATCCTGTCAAACACT |
| Hyg-R210 | | GCCGATGCAAAGTGCCGATAAACA |
| Hyg-F850 | | AGAGCTTGGTTGACGGCAATTTCG |
|  | |  |
| FgShy1-attb2r | | GGGGACAGCTTTCTTGTACAAAGTGGAACTATGGGTATCGCCTTGTCTTC |
| FgShy1-attb1r | | GGGGACTGCTTTTTTGTACAAACTTGTGAGATGCGTCCTTTGGAGTT |
|  | |  |
| FgShy1-attb4 | | GGGGACAACTTTGTATAGAAAAGTTGTTGTTCTTCCAAGCTCTCCATCTC |
| FgShy1-attb3 | | GGGGACAACTTTGTATAATAAAGTTGGCTGTTGCCGATGCATTTAG |
|  | |  |
| FgShy1-ORF5' | | ATGACACAAACATCTAGCAAGCA |
| FgShy1-ORF3' | | ATCTCACAAGACTTGGCAATCTC |
|  | |  |
| M13-For | | CGCCAGGGTTTTCCCAGTCACGAC |
| M13-Rev | | AGCGGATAACAATTTCACACAGGA |
| HY | | GGATGCCTCCGCTCGAAGTA |
| YG | | CGTTGCAAGACCTGCCTGAA |
| FgShyC-RO | | CATACTCTCACGGCCAACAA |
| FgShyC-RN | | GATGTTTCGTTGGTGACGGAG |
| FgShyC-RI | | GTCGTGACTGGGAAAACCCTGGCGGTGAATTTGCAAGGGTTGTAGG |
| FgShyC-LO | | CATCTCGTCTTCCATCCAGTTC |
| FgShyC-LN | | GCGTCAAATGATGACCAAGAAG |
| FgShyC-LI | | TCCTGTGTGAAATTGTTATCCGCTACTTGATTGGCTTGGTTACTG |
| FgShyC-RC | | CCTACAACCCTTGCAAATTCAC |
| FgShyC-RC | | CAGTAACCAAGCCAATCAAGT |
|  | |  |
